# Epidemiology and Species Distribution of *Candida* Bloodstream Infections in a Comprehensive Cancer Center, 2008–2023

**DOI:** 10.1007/s11046-026-01088-z

**Published:** 2026-09-02

**Authors:** Mohammad El-Atoum, George L. Chen, Brahm H. Segal, John P. Bonnewell, Nikolaos G. Almyroudis

**Affiliations:** 1 Department of Medicine, Jacobs School of Medicine and Biomedical Sciences, State University of New York at Buffalo, Buffalo, NY USA; 2 Stem Cell Transplantation and Cellular Therapy, University of Texas MD Anderson Cancer Center, Houston, TX USA; 3 Department of Internal and Hospital Medicine, H. Lee Moffitt Cancer Center and Research Institute, Tampa, FL USA; 4 Division of Infectious Diseases, Department of Internal Medicine, Roswell Park Comprehensive Cancer Center, Elm & Carlton Streets, Buffalo, NY 14263 USA; 5 Department of Pathology and Laboratory Medicine, Roswell Park Comprehensive Cancer Center, Buffalo, NY USA

**Keywords:** Epidemiology, Candidemia, *Candida* species, Solid tumors, Hematological malignancies

## Abstract

**Introduction:**

Candida bloodstream infections remain a significant complication of malignancies and their therapies.

**Aim:**

To investigate the epidemiology of *Candida* species causing bloodstream infections in patients with malignancies.

**Methods:**

We retrospectively reviewed bloodstream infections due to *Candida* from July 2008 to June 2023 in patients admitted under hematological, solid tumor and surgical services. Common species included *C. albicans, Nakaseomyces glabratus* (formerly *C. glabrata*), *Pichia kudriavzevii* (formerly *C. krusei*)*, C. parapsilosis* complex and *C. tropicalis*. The rest were classified as uncommon.

**Results:**

Incidence of candidemia was 0.46 episodes per 1,000 patients-days of admission. Of 265 episodes of candidemia, 102 (38.5%) occurred in patients under hematological services, 59 (22.3%) under solid tumor and 104 (39.2%) under surgical services. Among 271 available isolates, *C. albicans* was the predominant species, accounting for 96 (35.4%). *N. glabratus* (formerly *C. glabrata*) was the dominant species in 15 (55.6%) episodes of candidemia following triazole exposure (*p*: 0.008) and *C. parapsilosis* the dominant in 15 (34.9%) following echinocandin exposure (*p*: < .001). Candidemia due to uncommon *Candida* species occurred in 22 (8.3%) episodes for the total cohort and in 17 (16.7%) in patients with hematological malignancies (*p*: < .001). Multivariable logistic regression analysis identified neutropenia (OR 4.5 [95% CI 1.7–11.9]) as independent predictor of candidemia caused by uncommon species.

**Conslusion:**

Candidemia due to uncommon species occurred with higher frequency in patients with malignancies, with the highest rates observed among those with hematological malignancies.

## Introduction

Invasive candidiasis remains a significant complication of cancer and its care, with higher risk population those with hematological malignancies and recipients of hematopoietic stem cell transplantation (HSCT) [1,2]. In particular, patients with malignancies feature many of the risk and host factors for development of invasive candidiasis including neutropenia and mucositis, presence of central venous catheters, total parenteral nutrition, surgeries involving the gastrointestinal tract, prolonged antibiotic exposure and care in the intensive care unit [3].

Culture of blood or other biological specimens remain the gold standard for the diagnosis of invasive candidiasis. It can detect viable *Candida* with a limit of detection of ≤ 1–10 CFU/mL. However, cultures are characterized by lengthy turnaround time requiring 24 to 48 h for growth and at least additional 24 h for identification after subculture [4]. Moreover, sensitivity of cultures for candidemia is around 70% and for deep seated infections 30% [5]. To overcome these shortcomings molecular biology methods have been employed. Polymerase chain reaction (PCR) of *Candida* DNA is a non-culture-based method that can yield results in less than 6 h. PCR-based assays are highly sensitive detecting ≤ 1–2 CFU/mL [6]. Although these methods represent significant advances in the diagnostic field of invasive candidiasis, with the exception of some PCR platforms, they are designed to detect the 5 most common *Candida* spp., specifically *C. albicans, Nakaseomyces glabratus* (formerly *Candida glabrata*), *Pichia kudriavzevii* (formerly *Candida krusei*)*, C. parapsilosis* complex, and *C. tropicalis*.

Prompt initiation of antifungal therapy is crucial for a favorable outcome. The mainstay of treatment has historically been fluconazole or an echinocandin, with amphotericin B reserved for resistant isolates or severe forms of invasive disease [7]. Several new antifungal agents are currently under development.

A recent study that interrogated a large database of electronic health records from patients in the United States from 2009 to 2017, showed that the incidence of *Candida albicans, N. glabratus* (formerly *C. glabrata*)*, C. parapsilosis* complex*,* and *C. tropicalis* did not change overtime [8]. The same study demonstrated an increase to 6.4% in species other than the common ones. This study did not provide data on patients with malignancies. Therefore, we intended to describe the epidemiology of bloodstream infections due to *Candida* species and study the distribution of *Candida* species in our population which includes individuals with hematological and solid malignancies. We found that beyond the five most prevalent species, less common species are recovered with high frequency. Consequently, we identified risk factors associated with infections caused by *Candida* species other than the five most prevalent ones.

### Methods

We retrospectively reviewed all patients with candidemia among patients with hematological and solid malignancies. The study was approved by the Institutional Review Board. Informed consent was waived as the study included a review of electronic medical records and clinical microbiology data. The data were deidentified upon retrieval from the medical records. The study was conducted at Roswell Park Comprehensive Cancer Center, a 137-bed facility dedicated to cancer care in Buffalo, New York, USA.

### Patient Cohort

All positive blood cultures for *Candida* species were retrieved by reviewing the microbiology database. Only episodes of candidemia were included. Infections due to yeast other than *Candida* species were excluded. Episodes of candidemia were retrieved from July 2008 to June 2023 (total 15-year period).

We retrospectively reviewed the electronic medical records of patients with candidemia and extracted data on demographics and basic characteristics, including age, sex, underlying malignancy, history of transplant or cellular therapy, surgery involving the gastrointestinal tract the preceding 6 weeks (not including endoscopic procedures or feeding tube insertions), mucosal disruption (chemotherapy-induced mucositis, *Clostridium difficile* colitis, enterocolitis, gastrointestinal GvHD), total parenteral nutrition, neutropenia, graft vs. host disease (GvHD), and prior antifungal exposure.

### Definitions

Candidemia was defined as at least one positive blood culture from a patient with consistent clinical manifestations. Recurrent episode of candidemia refers to a new episode of candidemia caused by the same organism after documented clearance of the initial episode. Incidence of candidemia was estimated as number of new cases per 1,000 patient days of hospital admission. Prior antifungal exposure was defined as the administration of effective systemic antifungal therapy any time during the previous 30 days and was recorded as number of days of antifungal administration. Common *Candida* spp. was defined as the most frequently occurring species: *C. albicans, N. glabratus* (formerly *C. glabrata*)*, P. kudriavzevii* (formerly *C. krusei*)*, C. parapsilosis* complex*,* and *C. tropicalis*. Species other than these were considered uncommon. Neutropenia was defined as absolute neutrophil count of less than 1,000 cells/μL. Prolonged neutropenia was defined as an absolute neutrophil count < 1,000/µL persisting for ≥ 10 or more days.

### Antifungal Prophylaxis

Consistently during the study period, antifungal prophylaxis was administered to all patients with hematologic malignancies with prolonged neutropenia, recipients of autologous transplantation during the period of neutropenia, and recipients of allogeneic HSCT during neutropenia and while receiving steroids or tacrolimus and sirolimus [9,10]. Posaconazole was the most frequently used oral prophylactic antifungal agent throughout the study. Isavuconazole was introduced in 2015 and was used as an alternative when intravenous formulation was necessary. Micafungin was the main echinocandin used as an alternative to azoles. Liposomal amphotericin B was the lipid formulation of amphotericin B used in our institution. In patients with solid malignancies and those under surgical services fluconazole and micafungin were used preemptively or empirically in the presence of appropriate risk factors such as abdominal surgery and gastrointestinal leakage, in accordance with the guidelines of the Infectious Disease Society of America [7].

### Microbiological Methods

Blood culture processing was performed with the BacT/ALERT^®^ system (bioMérieux, Marcy-l’Étoile, France) until 2016, after which the laboratory transitioned to the BACTEC^®^ platform (BD Diagnostics, Sparks, MD, USA). Sabouraud dextrose agar was used by the clinical microbiology laboratory at our institution for initial isolation of yeast. CHROMID^®^ *Candida* agar (bioMerieux Inc., Durham, NC, USA) differential media was used to aid in identification during isolate workup. Final identification was performed with VITEK 2 automated system (bioMerieux Inc.) and yeast API^®^ (bioMerieux Inc.) until 2021, after which it was replaced by MALDI-TOF mass spectrometry (Bruker Daltonics, Billerica, MA, USA).

### Statistical Analysis

Descriptive statistics were used to summarize demographic and basic characteristics. Data were summarized using median (range) for continuous variables and absolute and relative frequencies (%) for categorical variables. Categorical variables were compared with the Chi-squared test with Fisher exact adjustment when appropriate, to test for independence. Recurrent episodes or more than one episode of candidemia from the same patient were analyzed as independent events. Univariable analysis and predictors of candidemia due to uncommon *Candida* species were determined by binary logistic regression analysis. Uncommon species were considered dependent variable and neutropenia, mucosal disruption, transplantation, prior exposure to antifungals, surgery including the gastrointestinal tract and total parenteral nutrition (TPN) were independent variables. Only statistically significant variables were included in the final model. *P*-values of < 0.05 were considered statistically significant. Analyses were performed using IBM SPSS Statistics 31 (IBM Corp., Armonk, NY, USA).

## Results

Over a 15-year period, 265 episodes of candidemia were recorded in 252 patients. In 6 episodes of candidemia, two distinct *Candida* species were isolated in each case, resulting in a total of 271 available *Candida* isolates. Demographics and basic characteristics are provided in Table 1. Eleven patients had 2 episodes of candidemia and 1 patient 3 episodes. Of these, 4 had recurrent candidemia episodes caused by the same species (three due to *Nakaseomyces glabratus* (formerly *C. glabrata*), one due to *Candida parapsilosis* complex). The remaining patients had more than one episode due to different *Candida* species.

**Table 1:** Demographics and clinical characteristics of patients with *Candida* bloodstream infections, Roswell Park Comprehensive Cancer Center, July 2008–June 2023

	Patients with *Candida* bloodstream infections (n = 252)
**Demographics**	
Age, median (range)	63 (8—93)
Sex (Female/Male) n, %	113 (44.8) / 139 (55.2)
**Service, n (%)**	
Hematological services	96 (38.1)
Leukemia service	40 (41.7)
Lymphoma service	22 (22.9)
Transplant and Cellular therapy service	34 (35.4)
Solid tumor services	56 (22.2)
Surgical oncology services	100 (39.7)
**Malignancy, n (%)**	
Hematological malignancies	
AA/ALL/AML	54 (21.4)
CML/MDS/MPD	7 (2.8)
HL/NHL/CLL	30 (11.9)
MM	6 (2.4)
Solid malignancies	
Head neck cancer	7 (2.8)
Lung cancer	18 (7.1)
Breast cancer	5 (2.0)
GI malignancies	65 (25.8)
Urologic cancer	27 (10.7)
Gynecologic malignancies	24 (9.5)
Sarcoma	5 (2.0)
Other solid malignancies^*^	4 (1.6)

^*^Other solid malignancies: Medulloblastoma, schwannoma, glioblastoma, carcinoma of unknown primary

AA: aplastic anemia, ALL: acute lymphocytic leukemia; AML: acute myelocytic leukemia; CLL: chronic lymphocytic leukemia; CML: chronic myelogenous leukemia; GI: Gastrointestinal; HL: Hodgkin lymphoma; HLH: Hemophagocytic lymphohistiocytosis; MDS: myelodysplastic syndrome; MM: multiple myeloma; MPD: myeloproliferative disease; NHL: non-Hodgkin lymphoma

The incidence of candidemia was 0.46 episodes per 1000 patients-days of admission. Incidence was 0.38 episodes per 1000 patients-days of admission for patients under hematological services, 0.56 in patients with solid malignancies managed under medical services, and 0.51 in patients managed under surgical services. Of 265 episodes of *Candida* bloodstream infections, 102 (38.5%) occurred in patients with hematological malignancies, 59 (22.3%) in patients managed under solid tumor services and 104 (39.2%) in patients managed under surgical services (Table 2). A total of 104 episodes of candidemia occurred in patients under surgical services. Of these, 50 episodes (48.1%) occurred within six weeks of surgery involving the gastrointestinal tract with the remainder having late surgical complications, surgical conditions requiring conservative management or had surgeries not involving the gastrointestinal tract.

**Table 2:** Risk factors for Candida bloodstream infections due to common and uncommon species, Roswell Park Comprehensive Cancer Center, July 2008–June 2023

Risk factors	*Candida* bloodstream infections caused by common *Candida* spp. (n = 243) (n, %)	*Candida* bloodstream infections caused by uncommon *Candida* spp. (n = 22) (n, %)	Risk estimate (95% CI),* p-value*
**Clinical services**			
Hematological services	85 (35)	17 (77.3)	6.32 (2.25–17.72), *p*: < .001
Solid tumor services	55 (22.6)	4 (18.2)	NS
Surgical services	103 (42.4)	1 (4.5)	0.65 (0.01–0.49) *p*: 0.001
**Risk factors**			
Surgery including GI tract	51 (21.0)	0 (0)	0.79 (0.74–0.84), *p:* 0.01
Total Parenteral Nutrition	70 (28.8)	6 (27.3)	*NS*
Mucosal disruption	61 (25.1)	9 (40.9)	*NS*
Neutropenia	53 (21.8)	14 (63.6)	6.*274* (2.49–15.75), *p:* < .001
Transplantation and Cell therapy	31 (12.8)	6 (27.3)	*NS*
Autologous HSCT	3 (1.2)	0 (0)	*NS*
Allogeneic HSCT	25 (10.3)	6 (27.3)	3.27 (1.17–9.11), *p: 0.04*
CAR-T cell therapy	3 (1.2)	0 (0)	*NS*
GvHD	16 (6.6)	2 (9.1)	*NS*
GI GvHD	14 (5.8)	1 (4.5)	*NS*
ICU care	63 (25.9)	10 (45.5)	*NS*
Prior antifungal exposure, median days (range)	0 (0–30)	6 (0–30)	− 5.77 (− 11.19 to − 0.35), *p:* 0.04
Triazole exposure, median days (range)	0 (0–30)	0 (0–26)	*NS*
Echinocandin exposure, median days (range)	0 (0–30)	5 (0–30)	-5.38 (-9.74 to -1.02), *p:* 0.02
Polyene exposure, median days (range)	0 (0–30)	0 (0–30)	*NS*

NS = not significant (*p:* > *.05*)

GI: Gastrointestinal; HSCT: Hematopoietic stem cell transplantation; CAR-T cell therapy: Chimeric antigen receptor T-cell therapy; GvHD: Graft versus host disease, ICU: Intensive care unit

Mucosal disruption: chemotherapy-induced mucositis, Enterocolitis, Pneumatosis coli, Clostridium difficile colitis

All 4 patients with recurrent episodes had hematological malignancies. Recurrent episodes occurred at a median of 47 days (range 40–260) following the original episode. Recurrent episodes occurred in the setting of gastrointestinal GvHD (n = 5) or neutropenia along with mucositis (n = 3). There was no clinical or radiological evidence of uncontrolled source in any of these cases. From 116 episodes of candidemia in which the central line was removed and was available culture data, line infection was confirmed in 31 (26.7%). Thirty-day mortality was 59.1% (13/22) in candidemia due to uncommon species compared with 35.4% (85/240) in candidemia due to common species (*p* = 0.049).

**Fig. 1:**
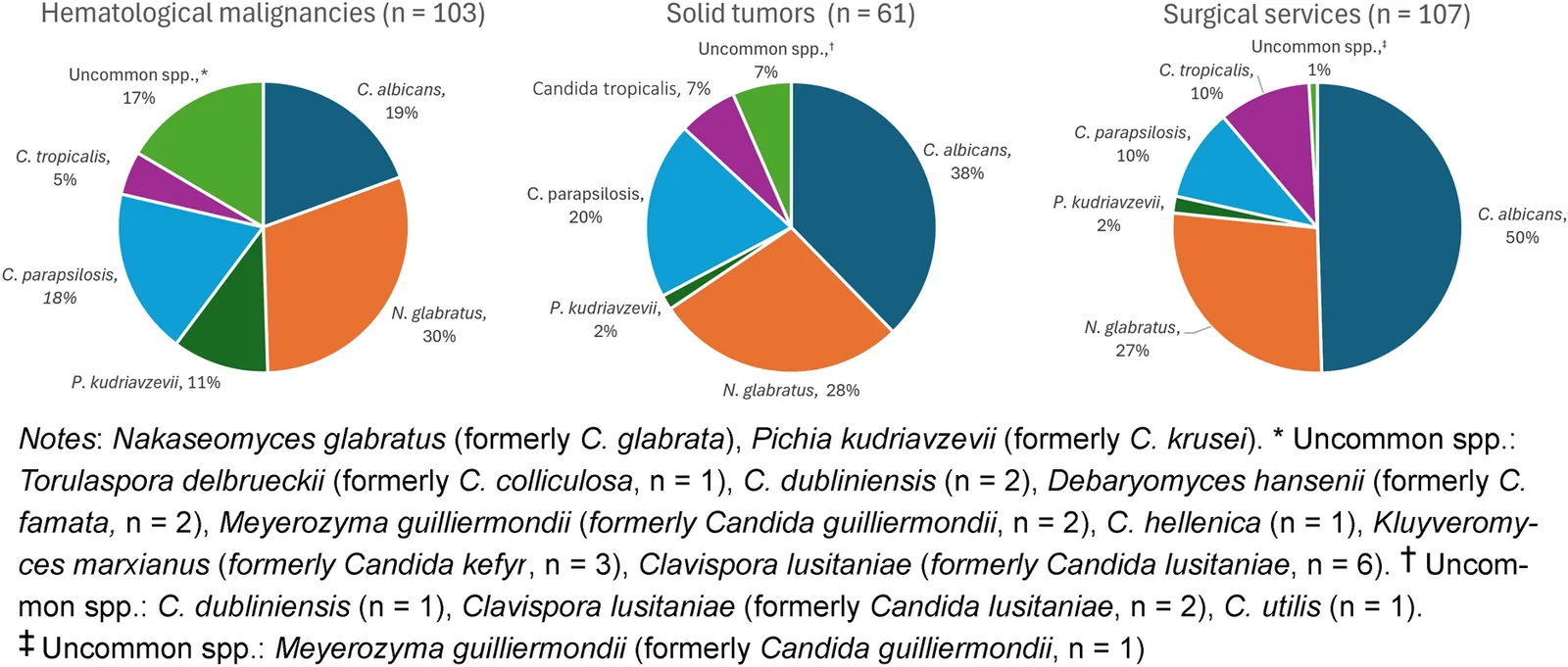
Distribution of *Candida* species across clinical services, Roswell Park Comprehensive Cancer Center, July 2008–June 2023

Among the 271 isolates, *Candida albicans* were the most prevalent species, accounting for 96 (35.4%), followed by *N. glabratus* (formerly *C. glabrata*) with 77 (28.4%), *C. parapsilosis* complex with 42 (15.5%), *C. tropicalis* with 20 (7.4%), and *P. kudriavzevii* (formerly *C. krusei*)*,* with 14 (5.2%). No *C. auris* was isolated in our cohort and no variation in species incidence was observed over time. The distribution of species within surgical and solid tumor services was similar, with *C. albicans* being the predominant species (n = 23, 37.7% and n = 53, 49.5% respectively) (Fig. 1). In patients with hematological malignancies, *N. glabratus* (formerly *C. glabrata*) was the predominant species (n = 31, 30.1%) followed by *C. albicans* (n = 20, 19.4%), *C. parapsilosis* (n = 19, 18.4%), *P. kudriavzevii* (formerly *C. krusei*, n = 11, 10.7%) and *C. tropicalis* (n = 5, 4.9%) with uncommon species cumulatively comprising a group of 17 cases of candidemia (16.5%). The ratio of *C. albicans* to non-albicans species was significantly lower in patients with hematological malignancies when compared to patients under solid tumor (*p*: 0.02) and surgical services (*p*: < 0.001), mainly driven by an increase in *P. kudriavzevii* (formerly *C. krusei*) (*p:* 0.007).

Of 271 available *Candida* isolates, 249 (91.9%) belonged to the 5 most common species, namely *C. albicans*, *N. glabratus* (formerly *C. glabrata*), *P. kudriavzevii*, *C. parapsilosis* and *C. tropicalis* (Fig. 1), while 22 (8.1%) were classified as uncommon species. Uncommon species included 8 (36.4%) *Clavispora lusitaniae* (formerly *Candida lusitaniae*), 3 (13.6%) *Meyerozyma guilliermondii* (formerly *Candida guilliermondii*), 3 (13.6%) *C. dubliniensis*, 3 (13.6%) *Kluyveromyces marxianus* (formerly *Candida kefyr*), 2 (9.1%) *Debaryomyces hansenii* (formerly *C. famata*), 1 (4.5%) *C. colliculosa*, 1 (4.5%) *C. utilis,* 1 (4.5%) *C. hellenica*. Of 265 episodes of candidemia, 22 (8.3%) episodes were caused by uncommon species, including 17 (16.7%) in patients under hematological services, 4 (6.8%) managed under medical solid tumor services and 1 (1.0%) under surgical services (*p:* 0.001). Although candidemia rates showed a downward trend over the entire study period, rates of candidemia due to uncommon species showed no variation over time [11].

A total of 97 (36.6%) of 265 episodes of candidemia developed following exposure to antifungals. This included 27 (10.2%) episodes with prior exposure to triazoles, 42 (15.8%) to echinocandins, 2 (0.8%) to liposomal amphotericin B, with another 26 (9.8%) having prior exposure to sequential or combination antifungal therapy. In 168 (63.4%) episodes, no prior antifungal exposure was documented. Prior antifungal exposure was significantly more frequent in patients with hematological malignancies compared to other services (*p:* < 0.001). In particular, prior antifungal exposure was documented in 79 of 102 (77.5%) episodes of candidemia in patients with hematological malignancies, 1 of 59 (1.7%) in solid tumor services, and 17 of 104 (16.3%) in episodes in surgical services.

**Fig. 2:**
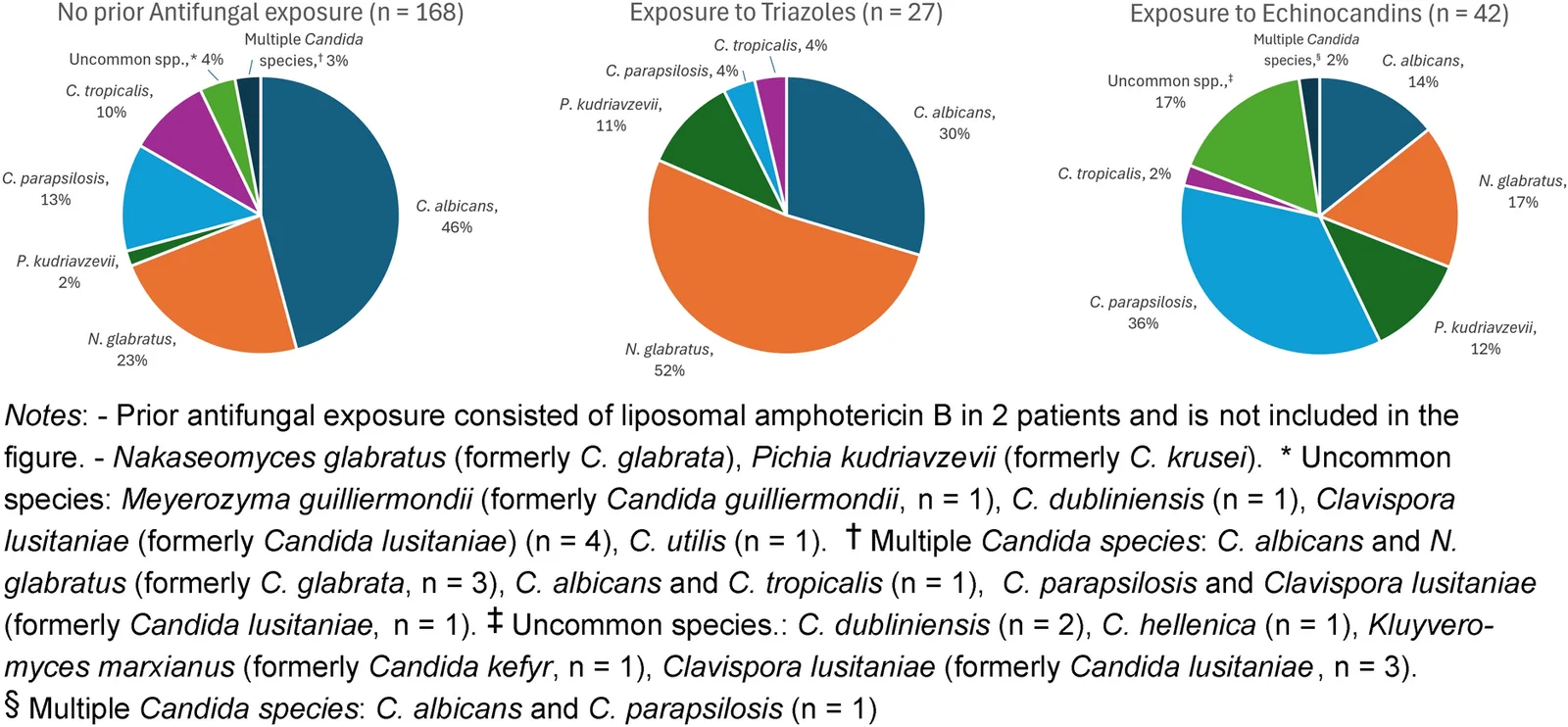
*Candida* bloodstream infection species distribution in relation to prior administration and class of antifungals, Roswell Park Comprehensive Cancer Center, July 2008–June 2023

Figure 2 illustrates the distribution of *Candida species* across *Candida* bloodstream infections in relation to prior administration and class of antifungals. *C. albicans* was the most common species in the absence of antifungal exposure, isolated in 78 (46.4%) episodes of candidemia. *N. glabratus* (formerly *C. glabrata*) was the dominant species isolated in 15 (55.6%) episodes following exposure to triazoles (*p*: 0.008) and *C. parapsilosis* in 15 (34.9%) episodes following exposure to echinocandins (*p*: < 0.001). Furthermore, any prior antifungal exposure was associated with a shift to non-albicans (*p:* < 0.001) and uncommon *Candida* spp. (*p:* 0.011).

Of the 22 isolates of uncommon *Candida* species, susceptibility data were available for 15. Fluconazole MIC range from 0.12 to 32 µg/mL with 11 isolates within the range of 0.12–2.0 µg/mL. Micafungin MIC ranged from 0.03 to 8 µg/mL with 12 isolates within the range of 0.12–1.0 µg/mL. All 15 isolates had MICs for amphotericin B within the range of 0.12–2.0 µg/mL.

Table 2 outlines the risk factors for bloodstream infections due to common and uncommon *Candida* species. Multivariable logistic regression model included surgery including the gastrointestinal tract, neutropenia, allogeneic HSCT and prior antifungal exposure. Multivariable logistic regression analyses identified neutropenia (*p:* 0.002, OR 4.5 [95% CI 1.7–11.9]) as independent risk factor for *Candida* bloodstream infection due to uncommon species.

## Discussion

Our study is a comprehensive investigation of the epidemiology of *Candida* species causing candidemia in patients with malignancies. We documented a sustained shift to non-*albicans* and uncommon *Candida* spp. primarily among patients with hematological malignancies.

*C. albicans* was the most common species in antifungal naïve patients and among patients with solid malignancies in our cohort, very similar το studies on general populations [2, 8, 12,13]. In contrast, a shift towards non-albicans and uncommon species was evident among patients with hematological malignancies when compared to patients under solid tumor (*p*: 0.02) and surgical services (*p*: < 0.001). Among common species this shift was primarily driven by *P. kudriavzevii* (formerly *C. krusei*) (*p:* 0.007), in contrast to prior reports identifying *N. glabratus* (formerly *C. glabrata*) as the most predominant species, likely reflecting local epidemiological variations [1,2, 14].

Shift to non-*albicans* species was significantly affected by prior antifungal exposure either as prophylaxis or treatment (*p:* < 0.001) [15,16]. Species selection was driven by the antifungal class. *N. glabratus* (formerly *C. glabrata*) was the dominant species in 15 (55.6%) episodes of candidemia following exposure to triazoles (*p*: 0.008) and *C. parapsilosis* complex in 15 (34.9%) following exposure to echinocandins (*p*: < 0.001) consistent with the literature [15, 17].

Incidence of uncommon species was significant in patients with hematological malignancies in our cohort. In the SENTRY Antifungal Surveillance Program (1997 to 2016) in which 20,788 invasive *Candida* isolates from 39 countries were analyzed, uncommon species corresponded to 3.2% of all isolates [12]. In the Prospective Antifungal Therapy (PATH Alliance^®^) study among 4,067 *Candida* isolates from patients with candidemia across multiple centers in North America (2004 to 2008), uncommon species accounted for 6.4% of all isolates [2]. In another study interrogating the health records of Cerner HealthFacts database in the United States (2009 through 2017), among 18,728 *Candia* isolates, uncommon species represented 6.4% of all isolates (also including *P. kudriavzevii*) [8]. In the ECMM Candida III study, a multicenter observational study evaluating candidemia across 20 European countries, among 399 isolates candidemia due to uncommon species occurred in 4.8% [18]. Findings from regional multicenter studies were comparable. Finally, in a cohort of 639 *Candida* isolates collected from 20 tertiary hospitals in South Korea, uncommon species accounted for approximately 5% [19]. Candidemia due to uncommon *Candida* species in our cohort accounted for 22 (8.3%) episodes for the total cohort, with a significantly higher proportion observed in patients with hematological malignancies (17 episodes, 16.7%; *p *< 0.001). The overall incidence was comparable, but the incidence in patients with hematological malignancies was remarkable.

These findings may have significant implications for the fungal diagnostic field, particularly with respect to the recently introduced molecular techniques. The principal advantages of PCR assays are the prompt identification of *Candida* at the species level independent of culture growth with a turnaround time up to 6 h and limit of detection of ≤ 5 CFU/mL [20]. Several PCR platforms have been developed to detect the most common *Candida* species including *C. albicans, N. glabratus* (formerly *C. glabrata*)*, P. kudriavzevii, C. parapsilosis* complex, and *C. tropicalis* [5, 20]. Moreover, T2Candida^®^, a fully automated platform that detects *Candida* species in whole blood by amplifying regions of the ribosomal DNA operon using a thermostable DNA polymerase combined with magnetic resonance, also included in the consensus definitions of invasive candidiasis [21], was designed to detect the 5 most common *Candida* spp. with the subsequent addition of *C. auris*. The striking incidence of 16.7% of uncommon species in patients with hematological malignancies in our cohort, makes the limited spectrum of the available PCR platforms suboptimal for this population.

Although univariable analysis identified allogeneic hematopoietic stem cell transplantation, neutropenia and prior antifungal exposure as associated conditions, only neutropenia remained an independent predictor in multivariable analysis. This finding is probably attributable to the inherently low virulence of uncommon *Candida* species and the presumed absence of effective immune evasive mechanisms.

Limitations of this study include its retrospective nature, single center data source, and the lack of data on sites of invasive candidiasis other than the bloodstream, source of candidemia and host factors. Due to the long study period, we were unable to fully account for alterations in cancer care, clinical practices and changes in host factors.

In conclusion the broader antifungal spectrum and superior safety profiles of newer triazoles and echinocandins made possible the widespread use of these agents as antifungal prophylaxis for patients at high risk [22]. Bloodstream infections due to uncommon *Candida* species occur with high frequency among patients with hematological malignancies. Neutropenia is independent predictors for bloodstream infections due to uncommon *Candida* species.

## Data Availability

The datasets generated during the current study are available from the corresponding author on reasonable request.
